# Mendelian randomization suggests causal correlations between inflammatory cytokines and immune cells with mastitis

**DOI:** 10.3389/fimmu.2024.1409545

**Published:** 2024-09-27

**Authors:** Jiaying Chen, Ben Su, Xinyue Zhang, Chao Gao, Yajie Ji, Xiaohong Xue

**Affiliations:** ^1^ Graduate School, Shanghai University of Traditional Chinese Medicine, Shanghai, China; ^2^ Department of Breast Surgery, Yueyang Hospital of Integrated Traditional Chinese and Western Medicine, Shanghai University of Traditional Chinese Medicine, Shanghai, China

**Keywords:** mastitis, cytokines, Mendelian randomization, genome-wide association study (GWAS), inflammation, immunity

## Abstract

**Objectives:**

Previous studies have reported that immunoinflammatory responses have associations with mastitis. Here, we aimed to further figure out whether circulating inflammatory cytokines and immune cells causally impact mastitis liability.

**Methods:**

The two-sample Mendelian randomization made use of genetic variances of 91 inflammatory cytokines from a large publicly available genome-wide association study (GWAS) containing 14,824 participants, 731 immunophenotypes data from 3,757 individuals as exposures separately, and mastitis from a GWAS summary (1880 cases and 211699 controls of European ancestry) as outcome. The primary analysis applied the inverse-variance weighted (IVW) method to estimate causal influences, with MR-Egger, weighted median, weighted mode and simple mode as supplementary approaches. Heterogeneity and pleiotropy were evaluated by the Cochrane Q test, MR-Egger intercept test, and MR-PRESSO global test.

**Results:**

The results indicated that CX3CL1 may be suggestively relevant to the risk of mastitis (odds ratio, OR = 1.434, 95% CI = 1.142~1.800, *p* = 0.002). Moreover, three immunophenotypes were identified as having a potential causal link to mastitis (*p* < 0.05). Significantly, CD28- CD8dim %CD8dim (OR = 1.058, 95% CI = 1.024 ~ 1.093, *p* = 0.0006) and CD45 on CD33br HLA DR+ (OR = 1.097, 95% CI = 1.039 ~ 1.157, *p* = 0.0008) were found to induce mastitis possibly. Conversely, CD39+ secreting Treg AC (OR = 0.929, 95% CI = 0.884~ 0.978, *p* = 0.005) pertained to protective factors of mastitis. Cochran’s Q test and MR-Egger intercept test indicated no significant heterogeneity (*p* > 0.05) or pleiotropy (*p* > 0.05), supporting the robustness and reliability of our findings.

**Conclusion:**

Our study adds to current knowledge on the causal roles of inflammatory cytokines and immune cells on mastitis by genetic means, thus guiding future clinical research.

## 1 Introduction

Mastitis is a painful benign breast disease that can be categorized into lactational and non-lactational mastitis. Lactational mastitis (LM) is the most common inflammatory disease of the mammary gland in primiparous women, usually occurring 3-4 weeks postpartum. Global incidence ranges from 3% to 33%, affecting breastfeeding and fetal health (1, 2). Mastitis can also occur in non-breastfeeding women, and rarely in men. Non-lactational mastitis is refractory including periductal mastitis (PDM) and granulomatous lobular mastitis (GLM) (2).

LM happens mostly due to milk stasis or nipple damage, bacteria that colonize the skin invade breasts along the lymphatic and milk duct, and subsequent infections in stagnant lactiferous ducts progress with Staphylococcus aureus being the main pathogen (3). Generally, 5% to 9% of reproductive-aged women will have PDM which often influences the subareolar ducts. The cause of periductal mastitis is still unclear while mammary ductal ectasia, plasma cell infiltration and abscess formation have been implicated as basic pathogenesis (4). In addition, more than one study shows immune responses may play a role (5). Alternatively, GLM is a rare autoimmune inflammatory disorder prone to recur that can be clinically similar to breast cancer with the highest risk in parous women ordinarily within 5 years of parturition (6). The etiology of GLM remains ambiguous while Corynebacterium infection may be closely related to the pathogenesis of GLM (7). The most widely accepted theory indicates that specific triggers such as trauma, bacteria, or exosmotic milk induce autoimmune destruction, giving rise to leakage of ductal secretions into the breast tissue and inflammatory cell infiltration, resulting in a granulomatous response. In such cases, patients may benefit from immunosuppressive therapy (8).

While clinical manifestations and etiology of these disorders are with great heterogeneity, inflammation and immune response appear to be the commonalities in the occurrence and development of such diseases, and their causal relationship remains to be verified. Mendelian randomization (MR) analyses aim to investigate causal correlations between exposures and outcomes via genetic variation such as single nucleotide polymorphisms (SNPs) as instrumental variables (IVs), making them less susceptible to environmental confounders and reverse causation (9). MR uses genetic variants as proxies for exposures to assess causal relationships, minimizing confounding and reverse causation. Although MR has been widely used to study the association between inflammatory factors and immune system disorders (10, 11), evidence on the causality of inflammatory cytokines and immune cells with inflammatory disorders of the breast remains limited. It is unclear whether they play a causal role or are merely a consequence of shared risk factors. However, the causal role of these factors in mastitis has not been fully established. To identify circulating inflammatory cytokines and immune cells as potential risk factors for mastitis, this study conducted a comprehensive two-sample Mendelian randomization (TSMR) analysis which explored their causal connection, providing insights into the role of inflammatory cytokines and immune cells in pathogenesis and potential therapeutic interventions of mastitis.

## 2 Materials and methods

### 2.1 Study design

**Figure 1:**
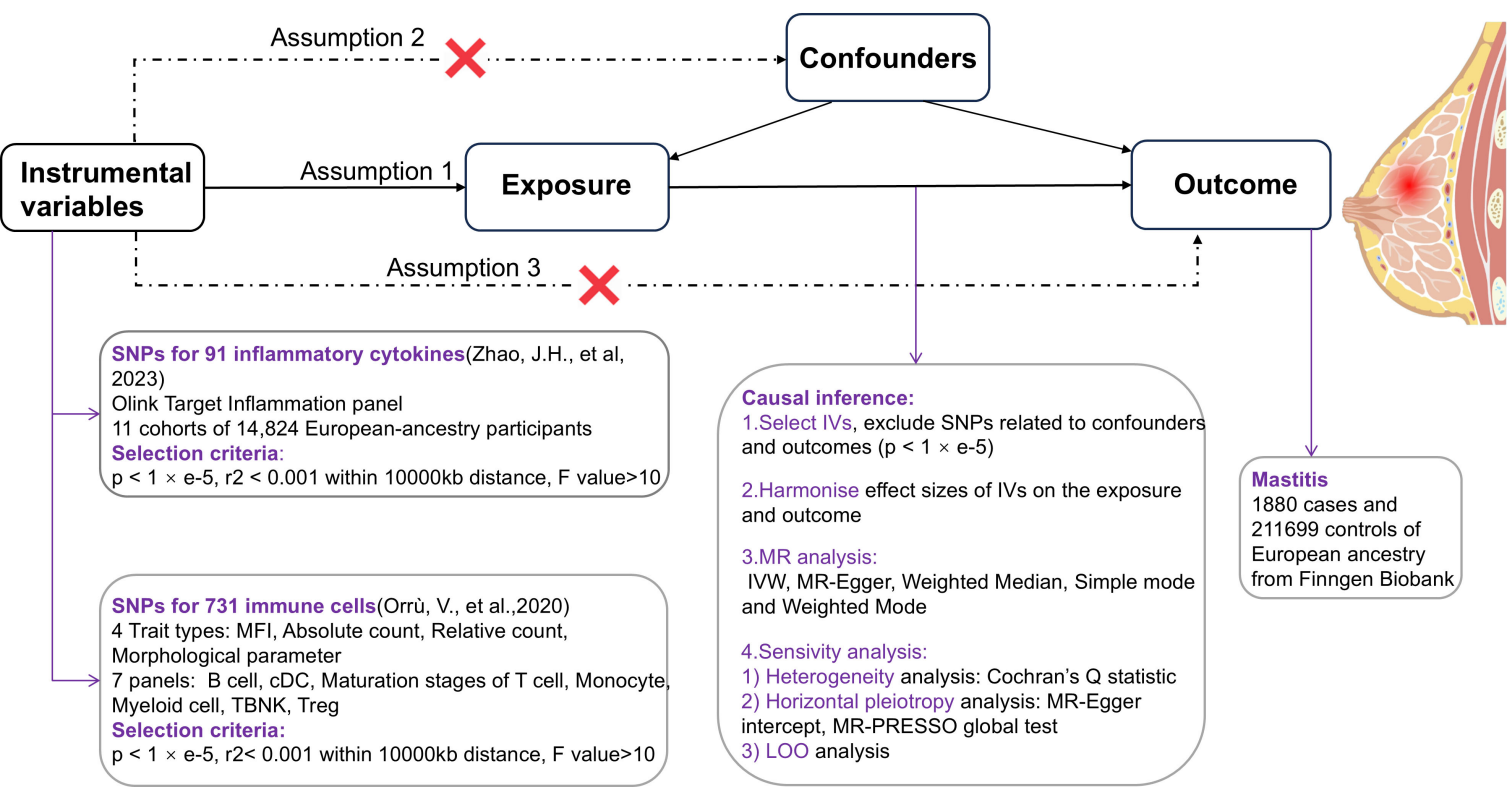
Schematic of the study design in TSMR.

This study employed a TSMR approach, with circulating inflammatory cytokines and immune cells as exposure factors separately, their genetic variant SNPs as IVs respectively, and mastitis as an outcome factor, to examine the causal relationship between exposure and outcome predicted by genes (12). In order to provide a reasonable interpretation of MR analyses, the following 3 core assumptions need to be met (13): ①Relevance - IVs are strongly associated with exposure factors, ②Independence - IVs are uncorrelated with confounders affecting both exposure and outcome, and ③Exclusivity - genetic variant SNPs affect outcomes solely through exposure (**Figure 1**).

### 2.2 Data sources

Genetic information on inflammatory factors is derived from the largest known meta-analysis of genome-wide association studies (GWAS) published in 2023 including 11 cohorts of total 14,824 European-ancestry participants (14). Results were meta-analyzed to identify significant protein quantitative trait loci (pQTLs) across 91 plasma proteins using the Olink Target Inflammation panel. 180 significant pQTLs involving 70 proteins were identified, with 33% of these being cis and 67% trans. Additionally, conditional analyses revealed 47 independent pQTL signals, increasing the total number of pQTLs from 180 to 227 (99 cis and 128 trans). Significant pQTL (*p ≤*5 × 10^−10^, fixed-effect meta-analysis) findings were validated in an independent cohort (ARISTOTLE) of 1,585 individuals. The analysis involved linear regression of cytokine levels against SNPs, adjusted for age, sex, and BMI, to identify single-variant associations (11). Besides, 72 out of 91 proteins were evaluated for replication in 35,556 participants in the deCODE study, using the aptamer-based SomaScan platform. Overall, 126 (71%) of the 178 testable pQTLs were replicated in either ARISTOTLE or deCODE. The data are publicly available at the website: https://www.phpc.cam.ac.uk/ceu/proteins and the EBI GWAS Catalog (accession numbers GCST90274758 to GCST90274848).

Summary statistics for 731 immune traits are also publicly available in the GWAS catalog database (accession numbers from GCST0001391 to GCST0002121). Among the 731 immune cells, 118 represent absolute cell counts, 389 reflect median fluorescence intensity (MFI), 32 are morphological parameters and 192 pertain to relative cell counts. The initial genome-wide association study on immune traits was conducted using data from 3,757 individuals of European descent, with no overlap between cohorts. A comprehensive set of approximately 22 million markers was genotyped using high-density arrays and subsequently imputed with a Sardinian sequence-based reference panel (15). The study discovered 122 independent signals for 459 cell traits at 70 loci (53 of them novel), revealing molecules and mechanisms involved in cell regulation with significant (*p* < 1.28 × 10^-11^) associations. Associations were assessed after adjusting for covariates such as sex, age, and age squared (16).

Genetic information of mastitis as an outcome factor comes from Finngen Biobank (https://r10.finngen.fi), which contains 1880 cases of inflammatory breast disease and 211699 controls of European ancestry (17).

### 2.3 Selection of instrumental variables

To further screen SNPs with strong correlative associations with inflammatory cytokines as exposed IVs, Firstly, we employed a loose genome-wide significant threshold to enhance the number of available SNPs (*p* < 1 × e-5) (18). Next, to exclude linkage disequilibrium (LD) and obtain independent IVs, we performed a clumping cutoff (r^2^ < 0.001 within a 10000-kb distance) (19). Meanwhile, SNPs strongly related to immune cells were selected when the following criteria were satisfied (*p* < 1 × e-5, r^2^< 0.001, kb=10000) (20). Then incompatible and palindromic SNPs were eliminated due to the inability to determine whether IV is oriented in the same direction during the harmonization process of the exposure and outcome data (21). After that, for the purpose of erasing the bias of weak IVs, the F value of each SNP was calculated by referring to the formula of F = beta^2^_exposure_/se^2^_exposure_ and R^2^ = 2× (1- MAF) ×MAF×beta^2^ (22) and finally, SNPs with F<10 were removed (23). Additionally, potential pleiotropic confounders were recognized by the Phenoscanner database (http://www.phenoscanner.medschl.cam.ac.uk/) (24).

### 2.4 Statistical analysis

The main analytical method used to determine the causal relationship between different circulating inflammatory factors and immune cells with mastitis is Inverse variance weighting (IVW) (25). MR-Egger, Weighted Median, Simple mode and Weighted Mode, as a supplement to IVW, can also be utilized to estimate causality (26, 27). It is worth noting that if these methods yield inconsistent results, we will prioritize IVW as the primary outcome. IVW is the major method commonly used in MR studies. It combines all the Wald ratios for each SNP to obtain a pooled estimate. Due to its robustness in providing consistent estimates when all instruments are valid, IVW was chosen as the primary method in this study. Potential heterogeneity was gauged by Cochran’s Q test with IVW and MR-Egger, and pleiotropy was assessed by the MR-PRESSO method and intercept of MR Egger test where all p values exceeding 0.05 signified the absence of both heterogeneity and pleiotropy (28, 29). Simultaneously, a Leave-one-out analysis was applied to evaluate the effect of a single SNP on sensitivity after removing SNPs one by one.

In this study, the odds ratio (OR) and 95% CI were presented. R version 4.3.2, R studio software and R package “TwoSampleMR (version 0.5.8)” were performed for the above analyses with the test criterion α = 0.05. To reduce the interference of correlation-level pleiotropy on the results, we searched the PhenoScanner V2 website to identify and rule out SNPs that were significantly associated (*p* < 1 × e-8) at the genome-wide level with shared risk factors for mastitis (hyperprolactinemia, nipple injury, smoking, bacterial infection et al.), and to re-run causal inference (1, 5, 30, 31).

## 3 Results

### 3.1 Influence of 91 inflammatory cytokines on mastitis

After selecting and coordinating IVs, we used a total of 30 SNPs for MR analysis. All SNPS had F statistics above 10 (ranging from 19.68 to 47.57) and were independent of relevant risk factors, suggesting that they were a powerful instrumental variable (The harmonized data are presented in **Supplementary Table 1**).

**Figure 2:**
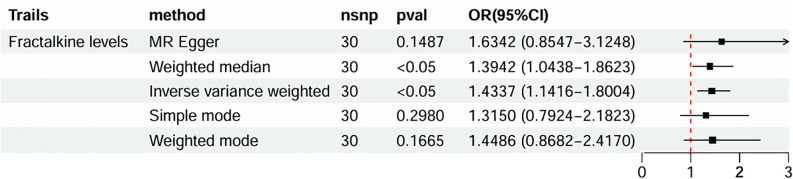
Forest plots of MR results of inflammatory cytokines on mastitis. MR, Mendelian Randomization; nsnp, number of SNPs; OR, odds ratio; CI, confidence interval.

**Figure 3:**
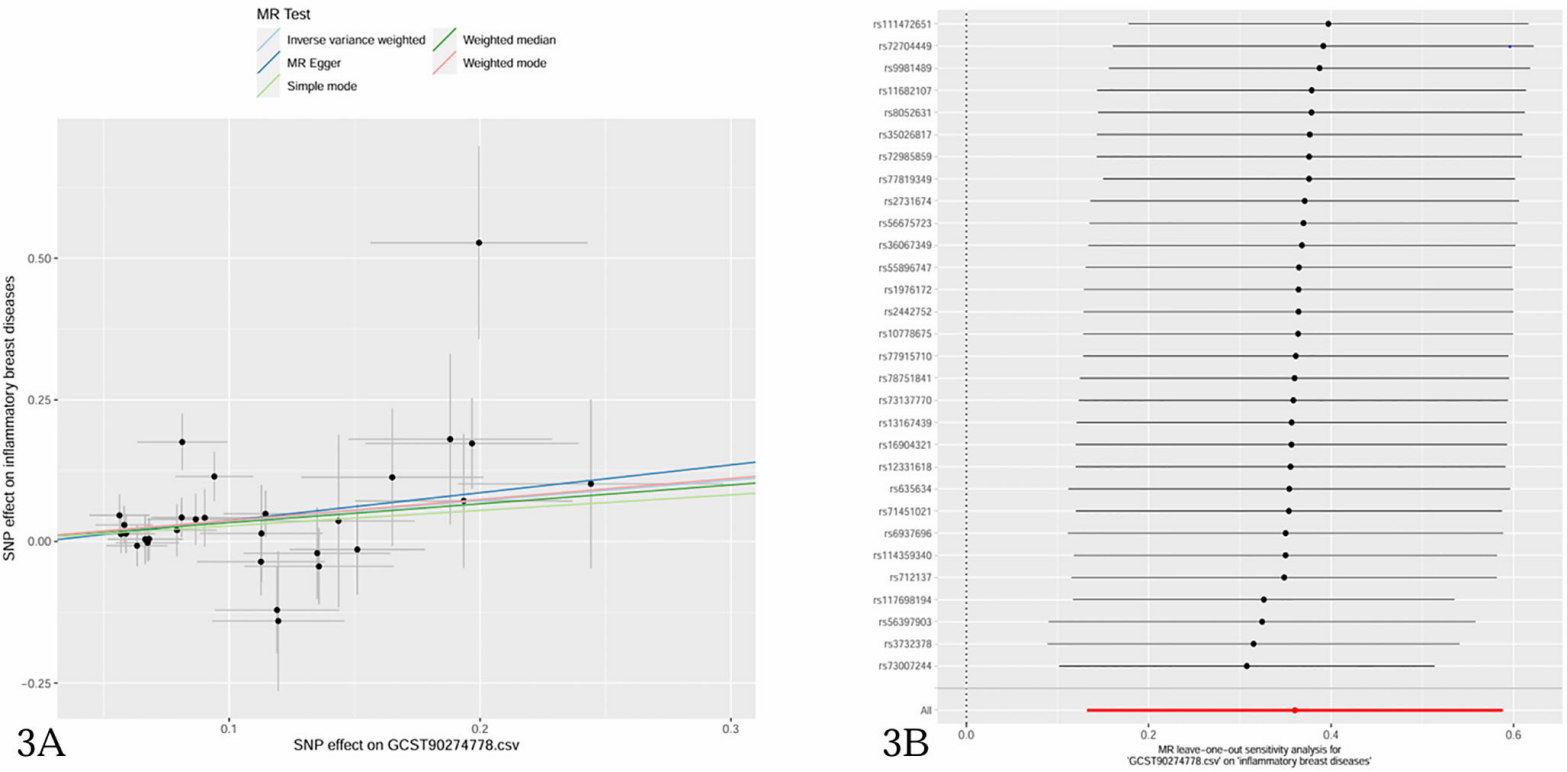
**(A)** Scatter plots of causal estimates of inflammatory cytokines (Fractalkine levels-CX3CL1) on mastitis. **(B)** Leave-one-out stability tests causal estimates of inflammatory cytokines (Fractalkine levels-CX3CL1) on mastitis.

The causal effect estimates of 91 circulating inflammatory cytokines on mastitis susceptibility are summarized in **Figure 2**. Employing IVW methodology as the primary analytical method, it was revealed that a genetically projected higher abundance of Fractalkine levels (OR: 1.434, 95% CI:1.142~1.800, *p* = 0.002) was linked to an increased mastitis risk and Weighted Median method also proved the trend (OR: 1.394, 95% CI:1.044~1.862, *p* = 0.024). **Figure 3A** illustrates the scatterplot depicting circulating inflammatory cytokines’ causal effects on mastitis. Meanwhile, the sensitivity analyses in this study did not provide any evidence of heterogeneity, based on Cochran’s Q test for CX3CL1 using the IVW (*p* = 0.156) or MR Egger (*p* = 0.133) methods (**Table 1**). Besides, the leave-one-out method did not identify cases where a single SNP significantly influenced the observed outcomes (**Figure 3B**). What’s more, as shown in **Table 1**, both the MR-Egger intercept test (*p* = 0.675) and the MR PRESSO global test (*p* = 0.176) confirmed the absence of horizontal pleiotropy.

**Table 1:** Results of the heterogeneity and horizontal pleiotropy for the effect of inflammatory cytokines on mastitis.

ID	Trait	Symbol	Heterogeneity	Pleiotropy		
			IVW CochraneQ pval	MR Egger intercept	MR Egger Intercept pval	MR PRESSO Global Test pval
GCST 90274778	Fractalkine levels	CX3CL1	0.156	-0.012	0.675	0.176

### 3.2 Influence of 731 immunophenotypes on mastitis

**Figure 4:**
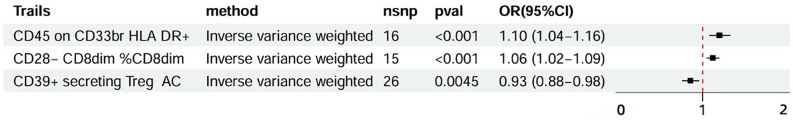
Forest plots of MR results of immune cells on mastitis. MR, Mendelian Randomization; nsnp, number of SNPs; OR, odds ratio; CI, confidence interval.

**Figure 5:**
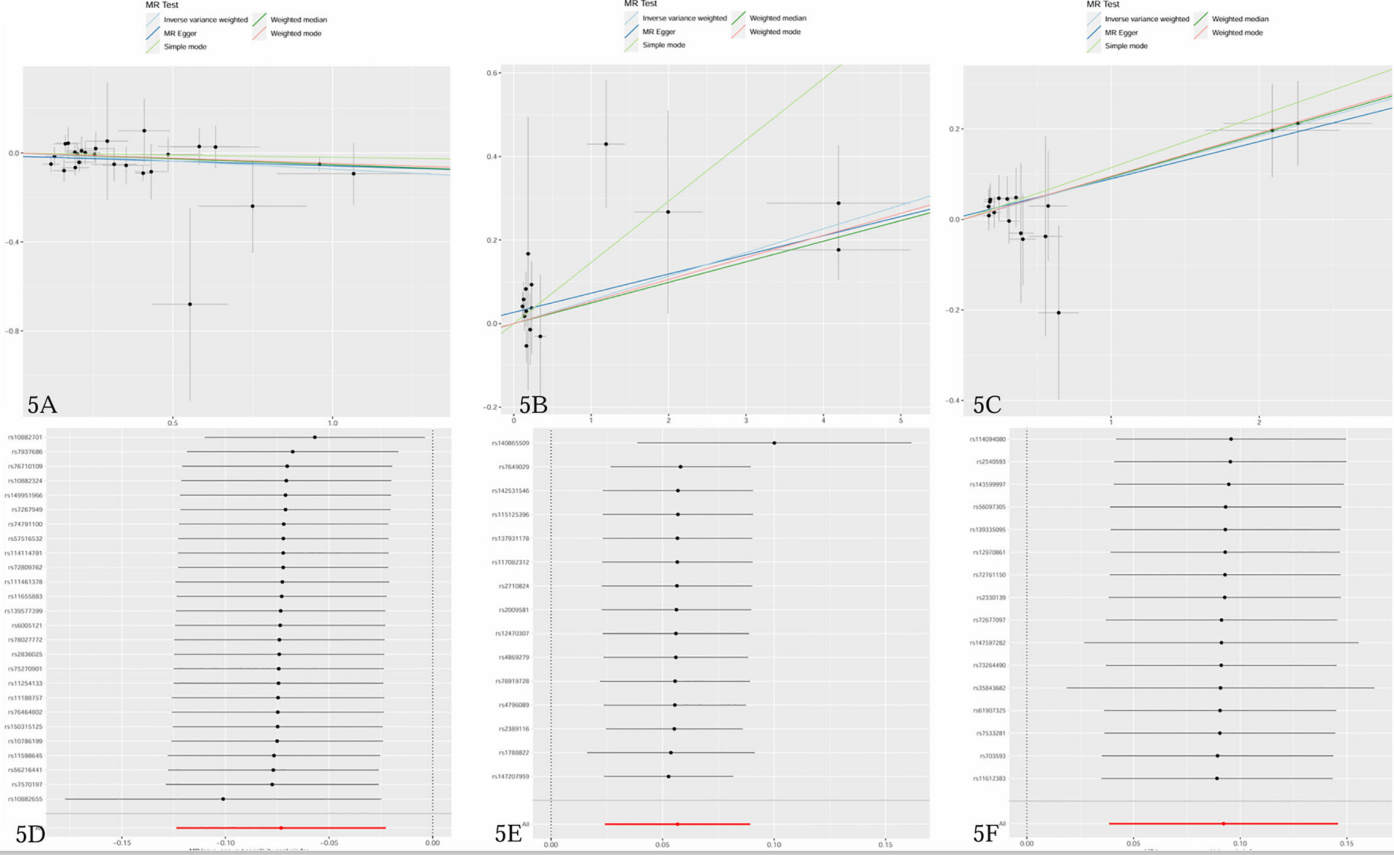
**(A–C)** Scatter plots of causal estimates of immune cells on mastitis. **(D–F)** Leave-one-out stability tests causal estimates of immune cells on mastitis. **(A, D)** CD39+ secreting Treg AC; **(B, E)** CD28- CD8dim %CD8dim; **(C, F)** CD45 on CD33br HLA DR+.

The study examined the causal effects of 731 peripheral immune cells on mastitis, as outlined in **Supplementary Table 2**. Utilizing 57 independent genome-wide significant SNPs as instrumental variables for immunophenotypes, with F-statistics ranging from 19.62 to 2198.81, Mendelian randomization analysis indicated that three immune cells exhibited potential causal relationships with mastitis (*p* < 0.05), with two showing an increased risk and one showing a negative association. The primary findings of the main Mendelian randomization analyses are depicted in **Figure 4**, while scatterplots illustrating the causal relationships of immune cells on mastitis are presented in **Figures 5A–C**. These three immune cells are distributed across three trait types and two panels: relative cell count (Treg panel), absolute cell count (Treg panel), and MFI count (Myeloid cell panel). The respective immunophenotypes were as follows. Strikingly, elevated levels of CD28- CD8dim %CD8dim (OR = 1.058, 95% CI = 1.024 ~ 1.093, *p* = 0.0006) and CD45 on CD33br HLA DR+ (OR = 1.097, 95% CI = 1.039 ~ 1.157, *p* = 0.0008) could induce the risk of mastitis. Consistent evidence supported the causal effects of CD28- CD8dim %CD8dim on mastitis across MR Egger (OR = 1.047, 95% CI = 1.013~ 1.082, *p* = 0.018), Weighted median (OR = 1.051, 95% CI = 1.013~ 1.090, *p* = 0.009) and Weighted mode methods (OR = 1.054, 95% CI = 1.017~ 1.092, *p* = 0.011). As to CD45 on CD33br HLA DR+, positive associations with a higher risk of mastitis were also observed by MR Egger (OR = 1.086, 95% CI = 1.014~ 1.163, *p* = 0.034), Weighted median (OR = 1.099, 95% CI = 1.016~ 1.188, *p* = 0.018) and Weighted mode methods (OR = 1.100, 95% CI = 1.027~ 1.179, *p* = 0.016). As shown in **Figure 5A**, genetically determined higher levels of CD39+ secreting Treg AC (OR = 0.929, 95% CI = 0.884~ 0.978, *p* = 0.005) were suggestively associated with lower odds of mastitis. Furthermore, neither heterogeneity nor pleiotropy was observed according to the results (**Table 2**). To detect whether any single instrument disproportionately influenced results, we conducted a leave-one-out analysis by systematically removing each SNP and repeating the MR. No high influence points driving the pooled IVW estimates were recognized in the Leave-one-out analysis (**Figures 5D–F**).

**Table 2:** Results of the heterogeneity and horizontal pleiotropy for the effect of immune cells on mastitis.

ID	Trait	Heterogeneity	Pleiotropy		
		IVW CochraneQ pval	MR Egger intercept	MR Egger Intercept pval	MR PRESSO Global Test pval
GCST90001495	CD39+ secreting Treg AC	0.813	-0.015	0.374	0.791
GCST90001662	CD28- CD8dim %CD8dim	0.209	0.027	0.115	0.412
GCST90002053	CD45 on CD33br HLA DR+	0.992	0.007	0.664	0.993

## 4 Discussion

In the two-sample MR analyses, we first conducted a thorough and comprehensive exploration of the causative links when circulating inflammatory cytokines and immune cells were seen as exposures separately and mastitis as the outcome, utilizing publicly accessible genetic data. And it was shown that CX3CL1, CD28- CD8dim %CD8dim and CD45 on CD33br HLA DR+ may suggestively be the upstream causes of mastitis, while heightened levels of CD39+ secreting Treg AC were connected to a reduced mastitis risk. Among immune cells, two risk factors were found to be related to the Treg panel (CD28- CD8dim %CD8dim) and Myeloid cells (CD45 on CD33br HLA DR+). Additionally, the presence of CD39+ secreting Treg AC from the Treg panel showed a negative association.

The mammary gland is a hormonally responsive exocrine gland. Immunoinflammatory response is a common phenomenon observed in individuals with mastitis (32). Also, it has been the subject of recent research providing compelling evidence of the crucial interplay between immunoinflammatory response with mastitis, including Liu, L., et al., noted that PDM patients exhibit a higher level of IFN- γ, and IL-12A compared to controls (5). These cytokines are secreted by T helper 1 cells aiding in eradicating foreign pathogens. Increased expression pattern of these cytokines suggests that immune responses may play a role in the pathogenesis of periductal mastitis. Similarly, the analysis of immune modulation by L. rhamnosus strains on mastitis identified two strains with significant anti-inflammatory potential, which can strongly induce IL-10 and weakly secrete pro-Th1 cytokine (IL-12 and IFN-γ). Therefore, these strains can be considered as a probiotic candidate for the management of infectious mastitis during lactation (33). After being exposed to lipopolysaccharide (LPS) *in vitro* experiments, untreated bovine mammary epithelial cells demonstrated a noteworthy reduction in the expression of CXCL8, IL1B, CCL20, and CXCL1 upon inflammatory response (34). Barreto, D.S., et al., also indicated autoimmune response is the most widely accepted etiology of GLM (35), and treatment with immunosuppression (steroids combined with Methotrexate) is an effective breast-conserving option. T lymphocyte subsets play a crucial role in maintaining immune homeostasis, with Treg cells exerting an immunosuppressive function. Likewise, Ucaryilmaz, H., et al., confirmed that there was an intrinsic defect of Tregs in patients with idiopathic granulomatous mastitis through flow cytometry. The expression of Foxp3 in Treg cells, and the frequency of non-suppressive Tregs, were found to be significantly lower in patients with idiopathic granulomatous mastitis when compared to healthy controls (36). However, different immunophenotypes in the Treg panel exhibited distinct roles in our results. Specifically, CD28- CD8dim %CD8dim was involved with increased risk while CD39+ secreting Treg AC demonstrated an opposing effect. Increasing evidence suggests that different subtypes of Tregs appear to have distinct roles in regulating the immune system. CD28 is a key co-stimulatory receptor typically expressed on most CD4+ T cells, binding CD80 and CD86 on antigen-presenting cells to promote T-cell activation. Lack of CD28 expression might impair their functional effectiveness or alter their activation state, leading to increased inflammation or reduced regulatory function. CD4+ CD28- T cells are associated with autoimmune diseases like rheumatic arthritis. Huang Y et al. also discovered that the absence of CD28 expression could identify T cell subsets exhibiting terminally differentiated and senescent characteristics, which were accompanied by the deterioration of the disease (37). Decreased CD28 expression can be an indicator of T-cell exhaustion. While CD39 is an ectoenzyme that converts ATP and ADP to AMP, it helps restrain excessive inflammatory responses (38). The immunoregulatory functions contribute to maintaining immune homeostasis and mitigating tissue damage. Chen C et al. demonstrated the immunosuppressive effect of CD39+ Tregs on acute lung injury via autophagy and the ERK/FOS Pathway (39). Our findings indicate a potential role of Tregs in the pathogenesis of idiopathic granulomatous mastitis and suggest a possible immunological basis for the development of this disease. These insights may inform future therapeutic approaches by targeting Treg cells and addressing the underlying immune dysregulation, which could generate novel therapies in the treatment and management of mastitis. Further investigation into the role of Tregs in this condition is warranted in order to develop more effective diagnostic and treatment strategies for patients with granulomatous mastitis. According to Zhao, J., et al., the expression of Th1 and Th17 cytokines was found to be significantly higher in the breast tissues of patients suffering from nonpuerperal mastitis, whereas the levels of Th2 cytokine and Treg cytokines such as IL-10 and transforming growth factor β were observed to be lower (40). In models of both sterile inflammation and bacterial infection, a diverse recruitment of myeloid cells was observed. The number of CD45+ leukocytes within the mammary gland significantly increased during inflammation and infection, which was in agreement with our studies. Among sterile inflammation models, increased numbers of neutrophils, Ly6C low monocytes and CD11b+F4/80+ macrophages were detected. While numbers of DCs and CD206+ macrophages increased among infection models, which was not seen during the sterile inflammation challenge. Our research also found that CD45 on CD33br HLA DR+ from Myeloid cells was linked to an increased risk of mastitis causally. These findings can provide valuable insights into the different immune responses of the mammary gland during inflammation and infection (41). Through flow cytometric analysis of milk, nonspecific mastitis revealed the highest percentage of CD4+ T lymphocytes, the percentage of CD8+ T lymphocytes was found to be highest in infectious bacterial mastitis (42). Our results showed CD28- CD8dim %CD8dim immunophenotype was significantly linked to the risk of mastitis (p = 0.0006). In the light of these observations, limited research on the relationship between immunoinflammatory response and mastitis may present a new perspective for early detection, prevention and monitoring. It is therefore necessary to conduct further research to explore the specific mechanism of these inflammatory cytokines and immune cells in mastitis.

To the best of our knowledge, this is the first Mendelian randomization research to evaluate the causal relationship between 91 inflammatory cytokines and 731 immune cells with mastitis, which provides genetic evidence emphasizing the strong causal relevance between immunoinflammatory response and mastitis. Nevertheless, it is crucial to acknowledge the restrictions of our study. Firstly, the obtained GWAS summary data predominantly consisted of patients of European descent, potentially generating biased estimates so it should be taken with caution if the conclusions would be applied in diverse ethnic groups. Future research should include diverse populations to determine whether these findings are consistent across different ethnic groups, ultimately enhancing the generalizability of the results and providing a more comprehensive understanding of genetic associations. Secondly, subgroup analyses aimed at attaining more precise correlations were not feasible due to the lack of specific demographic information and clinical records of study patients. Finally, owing to the limitations of the MR analysis, the second and third assumptions could not be properly examined and possible violations against these assumptions may exist. Colocalization analyses could be performed to further explain potential causal variation.

## 5 Conclusion

To sum up, our TSMR findings suggest that circulating inflammatory cytokines and immune cells might causally influence the risk of mastitis. It can be concluded an upregulation of CX3CL1, CD28- CD8dim %CD8dim and CD45 on CD33br HLA DR+ may contribute to an increased risk of mastitis, while raised levels of CD39+ secreting Treg AC are associated with a lower risk. Furthermore, these biomarkers may act as the initiatives in the onset and progression of mastitis, which could potentially serve as a viable approach for earlier diagnosis and more effective treatment options. They can also serve as candidate molecules for future mechanism exploration.

## Data Availability

The original contributions presented in the study are included in the article/**Supplementary Material**. Further inquiries can be directed to the corresponding author.
